# Total Neoadjuvant Therapy vs Standard Therapy in Locally Advanced Rectal Cancer: A Systematic Review and Meta-analysis

**DOI:** 10.1001/jamanetworkopen.2020.30097

**Published:** 2020-12-16

**Authors:** Anup Kasi, Saqib Abbasi, Shivani Handa, Raed Al-Rajabi, Anwaar Saeed, Joaquina Baranda, Weijing Sun

**Affiliations:** 1 Division of Medical Oncology, Department of Medicine, Kansas University Medical Center, Westwood; 2 Department of Internal Medicine, Icahn School of Medicine/Mount Sinai West and Morningside, New York, New York

## Abstract

**Importance:**

Standard therapy for locally advanced rectal cancer includes concurrent chemoradiotherapy followed by surgery and adjuvant chemotherapy (CRT plus A). An alternative strategy known as total neoadjuvant therapy (TNT) involves administration of CRT plus neoadjuvant chemotherapy before surgery with the goal of delivering uninterrupted systemic therapy to eradicate micrometastases. A comparison of these 2 approaches has not been systematically reviewed previously.

**Objective:**

To determine the differences in rates of pathologic complete response (PCR), disease-free and overall survival, sphincter-preserving surgery, and ileostomy between patients receiving TNT vs standard CRT plus A.

**Data Sources:**

MEDLINE (via PubMed) and Embase (via OVID) were searched from inception through July 1, 2020, for the following terms: *anal/anorectal neoplasms* OR *anal/anorectal cancer* AND *total neoadjuvant treatment* OR *total neoadjuvant therapy*. Only studies in English were included.

**Study Selection:**

Randomized clinical trials or prospective/retrospective cohort studies comparing outcomes in patients with locally advanced rectal cancer who received TNT vs CRT plus A.

**Data Extraction and Synthesis:**

Data regarding the first author, publication year, location, sample size, and rates of PCR, sphincter-preserving surgery, ileostomy, and disease-free and overall survival were extracted using Preferred Reporting Items for Systematic Reviews and Meta-analyses guidelines and pooled using a random-effects model.

**Main Outcomes and Measures:**

Rates of PCR, sphincter-preserving surgery, ileostomy, and disease-free and overall survival.

**Results:**

After reviewing 2165 reports, 7 unique studies including a total of 2416 unique patients, of whom 1206 received TNT, were selected. The median age for the patients receiving TNT ranged from 57 to 69 years, with 58% to 73% being male. The pooled prevalence of PCR was 29.9% (range, 17.2%-38.5%) in the TNT group and 14.9% (range, 4.2%-21.3%) in the CRT plus A group. Total neoadjuvant therapy was associated with a higher chance of achieving a PCR (odds ratio [OR], 2.44; 95% CI, 1.99-2.98). No statistically significant difference in the proportion of sphincter-preserving surgery (OR, 1.06; 95% CI, 0.73-1.54) or ileostomy (OR, 1.05; 95% CI, 0.76-1.46) between recipients of TNT and CRT plus A was observed. Only 3 studies presented data on disease-free survival, and pooled analysis showed significantly higher odds of improved disease-free survival in patients who received TNT (OR, 2.07; 95% CI, 1.20-3.56; *I*^2^ = 49%). Data on overall survival were not consistently reported.

**Conclusions and Relevance:**

The findings of this systematic review and meta-analysis suggest that TNT is a promising strategy in locally advanced rectal cancer, with superior rates of PCR compared with standard therapy. However, the long-term effect on disease recurrence and overall survival needs to be explored in future studies.

## Key Points

**Question:**

Is total neoadjuvant therapy (TNT) associated with improved outcomes when compared with standard concurrent chemoradiotherapy followed by surgery and adjuvant chemotherapy (CRT plus A) for locally advanced rectal cancer?

**Findings:**

In this systematic review and meta-analysis of 7 unique studies including 2416 unique patients, TNT was found to be associated with a significantly higher rate of achieving a pathologic complete response and disease-free survival compared with the standard CRT plus A approach. No significant difference was found in rates of sphincter-preserving surgery or ileostomy requirements between the 2 approaches.

**Meaning:**

Total neoadjuvant therapy was associated with improved pathologic complete response rates and has a potential disease-free survival advantage compared with the standard CRT plus A strategy in locally advanced rectal cancer.

## Teaser

This systematic review and meta-analysis assesses the differences in rates of pathologic complete response, disease-free and overall survival, sphincter-preserving surgery, and ileostomy between patients with locally advanced rectal cancer receiving total neoadjuvant therapy vs standard chemoradiotherapy followed by surgery and adjuvant chemotherapy.

## Introduction

Colorectal cancer remains a deadly disease with a projected 53 200 deaths in the US in 2020.^1^ Widespread use of a multimodality treatment strategy involving neoadjuvant chemotherapy with radiotherapy and subsequent total mesorectal excision for locally advanced rectal cancer (LARC) has improved survival. However, during the past decade, reduction in mortality has slowed for rectal cancer^1^ owing to a high rate of distant metastasis (29%-39%).^2^ Long-term analysis has shown that preoperative chemoradiotherapy results in persistent local control. Despite the adoption of adjuvant postoperative chemotherapy, patients are more than twice as likely to present with a distant recurrence rather than tumor regrowth at the primary site.^2,3^ This situation emphasizes the urgency of devising upfront treatment strategies aimed at controlling obscure micrometastases.

Total neoadjuvant therapy (TNT) is one such therapeutic strategy that incorporates chemotherapy with chemoradiotherapy antecedent to surgery.^4^ Total neoadjuvant therapy as an alternative treatment for LARC is now supported by the National Comprehensive Cancer Network.^5^ It has been postulated to offer advantages such as enhanced compliance with planned therapy, reduction in the tumor stage, and exposure to chemotherapy sooner in the disease course that targets occult micrometastases and can help assess chemosensitivity. Herein, we performed a systematic review and meta-analysis to compare the incidence of pathologic complete response (PCR), surgical organ preservation, and disease-free survival between the traditional concurrent chemoradiotherapy plus neoadjuvant chemotherapy (CRT plus A) approach vs TNT.

## Methods

### Design

This systematic review and meta-analysis was exempt from institutional review board approval based on Kansas University Medical Center criteria. The study was conducted in conformity with the Preferred Reporting Items for Systematic Reviews and Meta-analyses (PRISMA) recommendations.

### Literature Search Strategy

MEDLINE (via PubMed) and Embase (via OVID) were searched from inception through July 1, 2020. The search terms were as follows: *anal/anorectal neoplasms* OR *anal/anorectal cancer* AND *total neoadjuvant treatment* OR *total neoadjuvant therapy*. The appropriate Medical Subject Heading (MeSH) terms were combined in the search builder.

### Selection of Studies

Studies were chosen on the basis of the following criteria: (1) randomized clinical trials or prospective/retrospective cohort studies, (2) patients with LARC who underwent surgery, (3) intervention in trials was TNT vs CRT plus A, and (4) information on outcomes of PCR rate and any of the following if available: rates of sphincter-preserving surgery, ileostomy, or disease-free or overall survival. Studies beyond the inclusion criteria or originally published in a language other than English were excluded.

### Statistical Analysis

Data regarding the first author, publication year, location, sample size (including numbers of patients who received standard therapy and TNT), and rates of PCR, sphincter-preserving surgery, ileostomy, and disease-free and overall survival were extracted. A random-effects model with inverse variance (DerSimonian and Laird method) was applied.^6^ Heterogeneity was estimated using the inconsistency index and χ^2^ test. Two-sided *P* < .05 indicated significance. All statistical analyses were performed using RevMan software, version 5.3 (Cochrane Collaboration).

## Results

**Figure 1.**
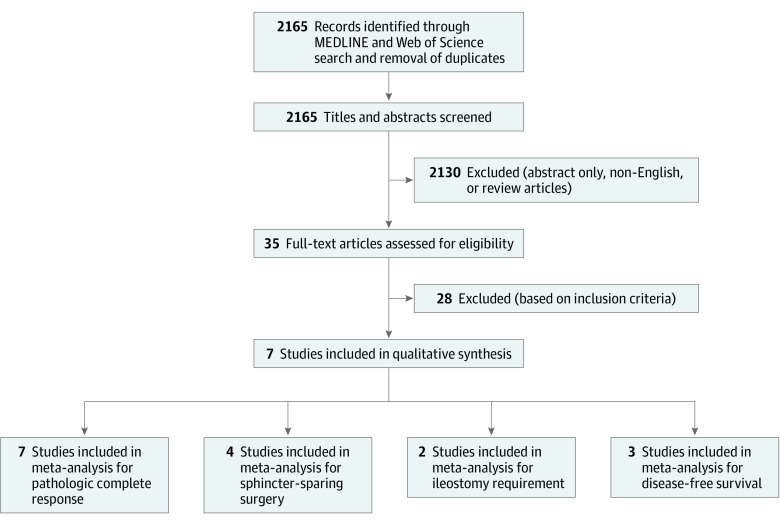
Study Flow Diagram A total of 7 studies including 2416 patients, of whom 1206 received total neoadjuvant therapy, were selected.

After reviewing 2165 reports, 7 unique studies^7,8,9,10,11,12,13^ that compared standard therapy and TNT were selected (Figure 1). These were reported from Europe and the United States and included a total of 2416 unique patients, of whom 1206 received TNT. The median age for the patients receiving TNT ranged from 57 to 69 years, with 58% to 73% being male. eTable 1 in the Supplement presents baseline characteristics of the studies included for analysis. Unfortunately, data on overall survival were not consistently reported.

**Figure 2.**
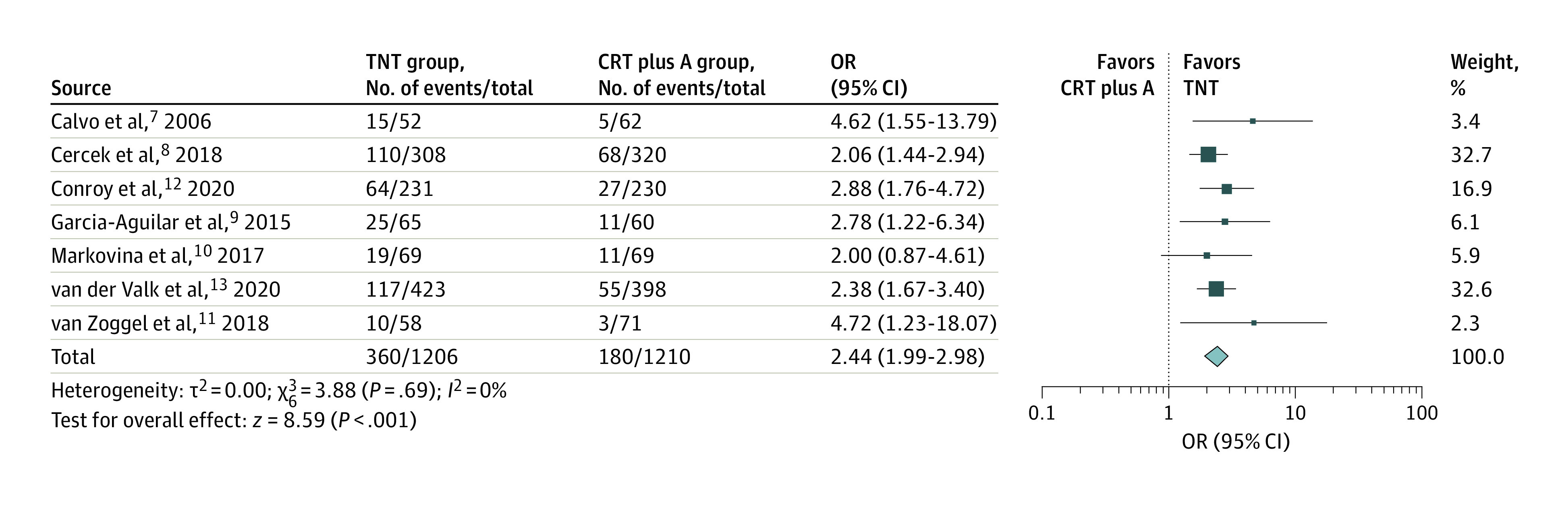
Forest Plot Comparing Proportion of Pathologic Complete Response Between Study Groups The total neoadjuvant therapy (TNT) and chemoradiotherapy followed by surgery and adjuvant chemotherapy (CRT plus A) groups were compared in a pooled analysis of randomized and nonrandomized trials. A random-effects model with inverse-variance method was used for the meta-analysis. OR indicates odds ratio; diamond, total OR; and marker size, weight.

The outcome of PCR was reported in all 7 studies. Patients in all studies underwent surgery apart from Cercek et al,^8^ in which a subset of patients who attained a clinical complete response (CCR) was observed. A cumulative metric of PCR for those who underwent surgery and sustained CCR (no evidence of local recurrence at 12 months) for those who did not undergo surgery was used. Figure 2 demonstrates the forest plot, with the pooled prevalence for PCR being 29.9% (range, 17.2%-38.5%) in the TNT group and 14.9% (range, 4.2%-21.3%) in the CRT plus A group. Total neoadjuvant therapy was associated with improved odds of attaining a PCR (odds ratio [OR], 2.44; 95% CI, 1.99-2.98). Separate analyses for randomized and nonrandomized studies are included in eFigures 1 and 2 in the Supplement.

**Figure 3.**
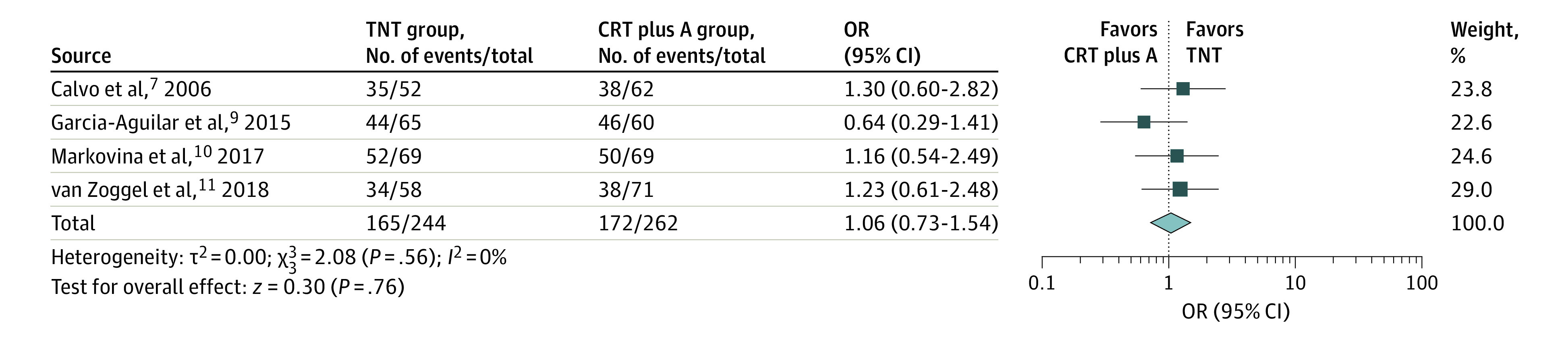
Forest Plot Comparing Proportion of Sphincter-Preserving Surgery Between Study Groups The total neoadjuvant therapy (TNT) and chemoradiotherapy followed by surgery and adjuvant chemotherapy (CRT plus A) groups were compared in a pooled analysis of randomized and nonrandomized trials. A random-effects model with inverse-variance method was used for the meta-analysis. OR indicates odds ratio; diamond, total OR; and marker size, weight.

Sphincter-preserving surgery was reported as a metric in 4 studies only.^7,9,10,11^ Rates of sphincter-preserving surgery did not significantly differ among the recipients of TNT vs CRT plus A (OR, 1.06; 95% CI, 0.73-1.54), as shown in the forest plot in Figure 3.

**Figure 4.**
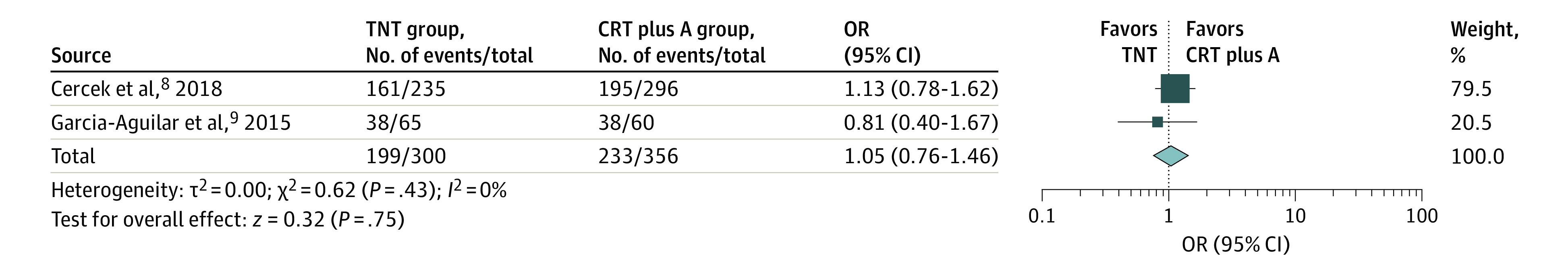
Forest Plot Comparing Proportion of Ileostomy Requirements Between Study Groups The total neoadjuvant therapy (TNT) and chemoradiotherapy followed by surgery and adjuvant chemotherapy (CRT plus A) groups were compared. A random-effects model with inverse-variance method was used for the meta-analysis. OR indicates odds ratio; diamond, total OR; and marker size, weight.

**Figure 5.**
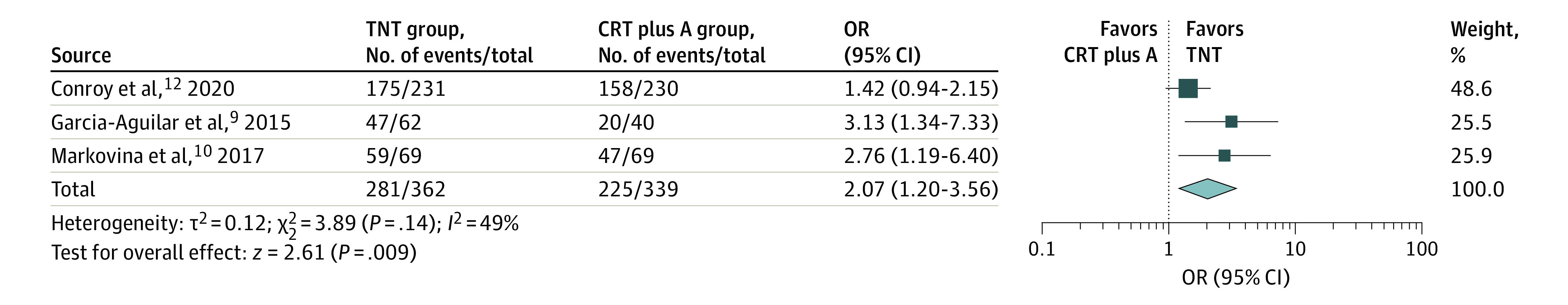
Forest Plot Comparing Disease-Free Survival Between Study Groups The total neoadjuvant therapy (TNT) and chemoradiotherapy followed by surgery and adjuvant chemotherapy (CRT plus A) groups were compared in a pooled analysis of randomized and nonrandomized trials. A random-effects model with inverse-variance method was used for the meta-analysis. OR indicates odds ratio; diamond, total OR; and marker size, weight.

Only 2 studies^8,9^ reported on ileostomy requirements (OR, 1.05; 95% CI, 0.76-1.46), with no statistically significant difference between both arms as shown in Figure 4. Although reported in only 3 studies,^9,10,12^ TNT was associated with a significantly longer disease-free survival. Taking into account the outcomes from these studies (Garcia-Aguilar et al^9^ described a 5-year disease-free survival whereas Markovina et al^10^ and Conroy et al^12^ reported a 3-year disease-free survival) generated an OR of 2.07 (95% CI, 1.20-3.56; *I*^2^ = 49%) (Figure 5) favoring improved disease-free survival among those who received TNT.^7,8,10^

## Discussion

This is the first systematic review and meta-analysis, to our knowledge, to compare the efficacy of TNT with that of conventional CRT plus A for LARC. The pooled analysis demonstrated a significantly higher chance of achieving a PCR as well as improved disease-free survival. Surgical outcomes, including rates of sphincter-preserving surgery and ileostomy, did not significantly differ among the 2 populations. The National Comprehensive Cancer Network guidelines already endorse the use of TNT; however, given that the current evidence is only preliminary, we sought to consolidate the evidence by performing a meta-analysis of relevant studies.

Pathologic complete response, determined by the lack of viable malignant cells in the surgically resected sample, is proposed as a pivotal prognostic criterion for long-term outcomes in LARC.^14^ Patients achieving PCR after neoadjuvant treatment are less likely to have a local tumor recurrence and more likely to have a better survival outcome than patients with an incomplete response.^15^ In a pooled analysis of survival outcomes for those attaining a PCR after preoperative chemoradiotherapy, 88.8% remained free of distant metastasis compared with 74.9% of patients without PCR at 5 years. Similarly, overall survival was 87.6% vs 76.4% for those with and without PCR, respectively, at 5 years.^16^ These results were replicated in the preoperative chemoradiotherapy arm of the German Rectal Trial (CAO/ARO/AIO-94 trial),^17^ wherein 86% of patients with a PCR were free of disease at the end of 5 years, compared with only 63% with an incomplete pathologic response. Whether adjuvant delivery of chemotherapy further augments this survival benefit in those who have already achieved PCR remains controversial.^18,19^ This finding emphasizes the need for developing better neoadjuvant strategies, such as TNT, that can enhance the rates of PCR and obviate the demand for postoperative chemotherapy in patients with a stoma, which is associated with lower adherence and greater toxic effects. A survey of the National Cancer Database^20^ revealed a 13% overall rate of PCR after conventional neoadjuvant CRT in all patients with rectal cancer, whereas our study demonstrated a cumulative rate of 29.9% for PCR after TNT.

Because a considerable reduction in the bulk of the tumor with a TNT approach might result in more and more patients adopting a nonoperative watch-and-wait strategy in the future, accurate determination of CCR in addition to PCR is imperative. The watch-and-wait strategy was introduced by Habr-Gama et al,^21^ who reported a highly promising overall survival of 97.7% and disease-free survival of 84% after a decade of follow-up in those who forgo surgery after attaining CCR. Another international multicenter registry-based study^22^ has also reported favorable outcomes for those with CCR who opt for the watch-and-wait approach, with a disease-specific survival rate of 94% with only 8% patients developing distant metastasis at 5 years. In a recent meta-analysis,^23^ patients opting for a watch-and-wait strategy after CCR to neoadjuvant chemoradiotherapy and patients with PCR identified at resection had no differences in terms of local recurrence or cancer-related mortality. The watch-and-wait approach may be deemed preferable because surgery can lead to bowel or bladder incontinence and sexual impairment as well as a short-term or permanent ostomy. A sustained CCR (at 1 year) was reported in 22% of those electing to forgo surgery in the retrospective analysis by Cercek et al (Memorial Sloan Kettering Cancer Center study).^8^

Van Zoggel et al,^11^ for the first time, evaluated TNT in patients with locally recurrent rectal cancer. The 3-year overall survival rate was 92% for patients who had a PCR, whereas it was only 54% in those without a PCR despite an R0 resection and 32% for those with an R1/R2 resection. The 3-year local recurrence–free survival as well as distant metastasis–free survival were also similarly increased for the patients who achieved PCR. Conroy et al^12^ more recently published data comparing a triple drug regimen of induction treatment with modified FOLFIRINOX (leucovorin [folinic acid], fluorouracil, irinotecan, and oxaliplatin) followed by CRT, with 3 months of adjuvant modified FOLFOX6 (leucovorin, fluorouracil, and oxaliplatin) or capecitabine compared with CRT with 6 months of adjuvant chemotherapy. The PCR rate was significantly higher in patients who received induction (27.5% vs 11.7%). Disease-free survival was also improved at 75.7% vs 68.5%, although the noted difference in this study compared with the previous studies is the inclusion of 3 months of adjuvant therapy. Three-year overall survival data have not yet matured.

Given that the chances of local recurrence are much reduced after PCR, the added value of surgery is questionable. Patients who undergo a sphincter salvage surgery are more likely to return to work and less likely to have sexual dysfunction or depression when compared with those who undergo abdominoperineal resection.^24^ Likewise, patients who opt for the watch-and-wait strategy have shown enhanced mental and physical recuperation and lesser disturbances with stooling, urination, or sex compared with those who undergo total mesorectal excision.^25^ Conroy et al^12^ have also reported a trend toward improved quality of life over time in the TNT group (*P* = .08) with lower rates of impotence. Although our meta-analysis does not highlight any specific surgical advantages in the TNT cohort, the Memorial Sloan Kettering Cancer Center study reported significant findings, such as earlier stoma closure (72% vs 9%) and a 25% higher rate of minimally invasive surgery in the TNT subset.^8^ Apprehensions regarding the safety of postponing surgery beyond 2 months after CRT were allayed by the TIMING (Timing of Rectal Cancer Response to Chemoradiotherapy Trial) trial.^9^ Although the rates of development of pelvic fibrosis were higher in the groups with a longer gap from CRT to surgery, they did not translate into an increased technical difficulty or postoperative complications.

A growing body of evidence^26,27^ suggests that surgery delayed for 3 or more months after radiotherapy is associated with a higher response rate compared with surgery performed within 12 weeks of radiation. In the study by Calvo et al,^7^ the TNT cohort underwent surgery about 1 month later than the preoperative CRT cohort, which may have contributed, in part, to the differences observed in the tumor downstaging. In the TIMING trial,^9^ a higher number of cycles of mFOLFOX6 as well as a longer gap between CRT and surgery were both independently associated with a PCR. In the Memorial Sloan Kettering Cancer Center study,^8^ a higher complete response rate (cumulative metric of PCR and CCR) still persisted in the TNT cohort after adjusting for the time lapse between CRT and surgery. None of the trials reported an increased tumor progression, despite the time to operative therapy being delayed by administration of neoadjuvant systemic chemotherapy. Lengthening the interval between short-course radiotherapy and surgery has also been shown to induce greater tumor downsizing with higher PCR rates (10.4% vs 2.2%) compared with the traditional preoperative long-course radiotherapy approach.^28^ Based on this premise, the landmark RAPIDO (Rectal Cancer And Pre-operative Induction Therapy Followed by Dedicated Operation) trial^13^ reversed the TNT order and compared short-course radiotherapy followed by neoadjuvant CAPOX (capecitabine and oxalipltin) or FOLFOX with subsequent total mesorectal excision after approximately 6 months with the preoperative long-course CRT followed by total mesorectal excision and optional adjuvant CAPOX or FOLFOX. The short-course radiotherapy group showed doubling of the PCR rate (28% vs 14%; *P* < .001) and a 7% higher distant metastasis–free survival at 3 years (26.8% vs 20%; *P* = .005), with comparable toxic effects and no increase in surgical or postoperative complications. However, the 3-year overall survival was similar in each arm at 89%. Even after excluding the time-interval factor, short-course radiotherapy has previously been shown to have similar rates of overall survival, local recurrence, or toxic effects compared with conventionally fractioned preoperative long-course CRT.^29^ Moreover, short-course radiotherapy provides a lesser dose of pelvic radiation and is more convenient and tolerable for patients.

Total neoadjuvant therapy can be administered exclusively as induction chemotherapy before CRT or in a consolidative manner after CRT. Preliminary results from the OPRA (Organ Preservation of Rectal Adenocarcinoma) trial^30^ showed a significantly higher rate of organ preservation in the consolidative TNT arm compared with the induction TNT arm but no significant difference when it came to 3-year disease-free or distant metastasis–free survival. Total neoadjuvant therapy has also emerged as a platform for investigating novel radiation sensitizers and systemic chemotherapy and immunotherapy agents in LARC. The ongoing NRG-GI002 trial^31^ is a multiarm randomized phase 2 clinical trial that is using TNT for testing parallel experimental arms. Currently, veliparib and pembrolizumab are being evaluated in conjunction with total neoadjuvant CRT, whereas other experimental arms are in development. In addition, immune checkpoint inhibitors such as durvalumab, avelumab, and nivolumab and other novel agents such as peposertib, a DNA protein kinase inhibitor, and aflibercept, an angiogenesis inhibitor, are being investigated in a TNT setting.^32,33,34,35,36^ A list of recent and ongoing TNT trials that are yet to be published is included in eTable 2 in the Supplement.^30,31,32,33,34,35,36,37,38^

Identifying predictive biomarkers for patients who are more likely to benefit from TNT is a current need. A National Cancer Database study^39^ looked at more than 5000 patients and reported a significantly reduced rate of PCR after neoadjuvant CRT in patients with microsatellite instability (stability vs instability, 8.9% vs 5.9%; OR, 0.65). However, a recent meta-analysis including 5 studies with 5800 patients^40^ demonstrated no significant difference in PCR between microsatellite instability or stability in LARC (10.1% vs 6.6%; OR, 1.38; *P* = .35). Another unmet need is the development of a reliable surrogate end point for clinical trials, because 3- or 5-year survival rates pose financial and practical challenges in the implementation of novel agents. One such surrogate end point for prognostication in patients treated with TNT is the neoadjuvant rectal score. The neoadjuvant rectal score is based on clinical T stage and pathological T and N stages and takes into account the effect of neoadjuvant therapy in downsizing the tumor. Multiple ongoing TNT trials are now including the neoadjuvant rectal score as a primary or secondary end point.^32,37^ In the CAO/ARO/AIO-04 trial,^41^ the neoadjuvant rectal score was found to be an independent predictor for disease-free, overall, and distant metastasis–free survival as well as local recurrence; however, a recent Netherlands Cancer Registry–based study including more than 6500 patients^42^ found the neoadjuvant rectal score to be poorly concordant with the true end point when compared with a simple Cox proportional hazards regression model using the same 3 criteria included in the neoadjuvant rectal score formula.

Owing to a more intensive neoadjuvant chemotherapy regimen, grade 3 or 4 toxic effects, such as neutropenia and lymphopenia, were more commonly reported in the TNT cohorts in 2 of the 7 studies.^9,10^ However, this did not translate into increased treatment discontinuation or dose reduction in the TNT cohorts. On the contrary, TNT is associated with better compliance rates than adjuvant chemotherapy.^8,10^ Grade 3 peripheral neuropathy was reported in 11.7% and 4% of patients in the TNT arm in the PRODIGE 23 (Partenariat de Recherche en Oncologie Digestive) trial^12^ and RAPIDO trial,^13^ respectively. Toxic effects to the gastrointestinal tract were reported to be higher in the CRT cohort in the study by Calvo et al,^7^ whereas the RAPIDO trial^13^ documented a much higher rate of grades 3 to 4 diarrhea in the TNT cohort (18% vs 7%). However, at 6 months, there was a significant increase in all grade 3 to 4 toxic effects, such as neutropenia, thrombocytopenia, lymphopenia, fatigue, diarrhea, anorexia, weight loss, and peripheral neuropathy in the CRT arm in the PRODIGE 23 trial,^12^ which concluded that for the same duration of chemotherapy, the preoperative approach was better tolerated than adjuvant therapy.

### Limitations

One of the major limitations of this study is that apart from PCR, none of the end points were consistently reported across all the 7 studies included in our meta-analysis, with only 4 studies^7,9,10,11^ reporting rates of sphincter-preserving surgery, 2 studies^8,9^ reporting ileostomy requirements, and only 3 studies^9,10,12^ reporting disease-free survival (eTable 1 in the Supplement). Likewise, insufficient data were available to calculate a pooled overall survival. Another significant metric, CCR, was reported in only 1 study^8^ and hence could not be meta-analyzed. Another limitation is that the studies incorporated in our analysis were a mix of randomized and nonrandomized trials. However, we addressed this issue by conducting separate analyses for randomized and nonrandomized studies and then pooling the results.

## Conclusions

Total neoadjuvant therapy appears to be a promising treatment strategy that has been reported in several trials. Total neoadjuvant therapy enhances one’s chances of attaining a PCR, which traditionally has been shown to correspond to higher overall and disease-free survival. Our meta-analysis suggests an improved disease-free survival, although the true effect of TNT on overall and disease-free survival is unclear and requires further evaluation in a prospective randomized manner.

Total neoadjuvant therapy theoretically offers multiple surgical advantages, such as improved odds of receiving a sphincter-sparing surgery and lower odds of requiring an ileostomy; however, neither of these outcomes was evident in our meta-analysis, suggesting that the benefit is primarily in disease control and decreased recurrence rates. Because we might be moving toward an era of a watch-and-wait approach in patients who are able to demonstrate sustained CCR, several questions, such as the reliability with which patients with a CCR can be identified, the optimal diagnostic test for monitoring of these patients in terms of imaging or gene expression profiling, and the appropriate follow-up duration, remain to be answered. Further trials are also needed for head-to-head comparisons of induction vs consolidative vs combined induction-consolidative TNT approaches. Advantages of TNT also need to be balanced with overadministration of cytotoxic therapy and long-term toxic complications in patients with low-risk disease. Future studies must also devise biomarkers to identify the patient cohort most likely to benefit from TNT.

Preoperative chemoradiotherapy has primarily been proven to be effective in local disease control but is not as successful in preventing distant metastasis, which has emerged as the primary mode of recurrence in rectal cancer. In light of this finding, early administration of systemic chemotherapy can potentially achieve long-term disease control and a positive prognostic effect.

## Notes

Supplement. **eTable 1.** Baseline Characteristics of the Studies Included in the Meta-analysis **eTable 2.** List of Ongoing/Recently Reported TNT Trials Other Than Those Included in the Meta-analysis **eFigure 1.** Comparison of PCR Rates in TNT vs CRT Plus A (Meta-analysis of Nonrandomized Studies) **eFigure 2.** Comparison of PCR Rates in TNT vs CRT Plus A (Meta-analysis of Randomized Studies)
