# Supplementary material for: Total Neoadjuvant Therapy vs Standard Therapy in Locally Advanced Rectal Cancer: A Systematic Review and Meta-analysis
Source: JAMA Netw Open. 2020 Dec 16;3(12):e2030097. doi: 10.1001/jamanetworkopen.2020.30097 (PMC7745099; doi:10.1001/jamanetworkopen.2020.30097)
Supplement: Supplement. — eTable 1. Baseline Characteristics of the Studies Included in the Meta-analysis eTable 2. List of Ongoing/Recently Reported TNT Trials Other Than Those Included in the Meta-analysis eFigure 1. Comparison of PCR Rates in TNT vs CRT Plus A (Meta-analysis of Nonrandomized Studies) eFigure 2. Comparison of PCR Rates in TNT vs CRT Plus A (Meta-analysis of Randomized Studies) [file jamanetwopen-e2030097-s001.pdf]

Supplementary Online Content

Kasi A, Abbasi S, Handa S, et al. Total neoadjuvant therapy vs standard therapy in locally advanced rectal cancer: a systematic review and meta-analysis. *JAMA Netw Open*. 2020;3(12):e2030097. doi:10.1001/jamanetworkopen.2020.30097

eTable 1. Baseline Characteristics of the Studies Included in the Meta-analysis

eTable 2. List of Ongoing/Recently Reported TNT Trials Other Than Those Included in the Meta-analysis

eFigure 1. Comparison of PCR Rates in TNT vs CRT Plus A (Meta-analysis of Nonrandomized Studies)

eFigure 2. Comparison of PCR Rates in TNT vs CRT Plus A (Meta-analysis of Randomized Studies)

This supplementary material has been provided by the authors to give readers additional information about their work.

©2020 Kasi A et al. *JAMA Network Open*.

eTable 1. Baseline Characteristics of the Studies Included in the Meta-analysis

Reference	Year	Study Interval	Number of patients	Outcomes reported	Study type
Calvo *et al*[8]	2006	1998-2002	114	PCR / SPS	Prospective cohort analysis
Cercek *et al*[9]	2018	2009-2015	628	PCR* / IR	Retrospective cohort analysis
Garcia-Aguilar *et al*[10]	2015	2004-2012	125	PCR / SPS / IR / DFS	Randomized Phase II
Markinova *et al*[11]	2017	2009-2012	138	PCR / SPS / DFS	Randomized Phase II – subset analysis
Van Zoggel *et al*[12]	2018	2010-2016	129	PCR / SPS	Retrospective cohort analysis
Conroy *et al***[13]	2020	2012-2017	431	PCR / DFS	Randomized Phase III
Van der Valk *et al*[14]	2020	2011-2016	821	PCR***	Randomized Phase III

PCR- Pathologic complete response, SPS- sphincter-sparing surgery, IR- ileostomy requirement, DFS-disease-free survival.

* Not all patients went to surgery, the collected metric is a combination of PCR for those who went to surgery and sustained clinical complete response rate of those who had no evidence of local recurrence at 12 months without surgery.

** Included mFOLFIRINOX for induction chemotherapy, TNT group received adjuvant 3 months of mFOLFOX6, CRT+A received 6 months of adjuvant chemotherapy.

*** TNT arm included short course radiotherapy followed by chemotherapy with CAPOX / FOLFOX4.

©2020 Kasi A et al. *JAMA Network Open*.

eTable 2. List of Ongoing/Recently Reported TNT Trials Other Than Those Included in the Meta-analysis

Study	Approach	Participants	End points	Results	Conclusion
**Garcia-Aguilar et al, 2020-OPRA trial** [31]	Pts with LARC were randomized to 4 months of FOLFOX or CAPEOX before (Induction) or after (Consolidation) fluorouracil or capecitabine based chemoradiotherapy (CRT). At 8-12 weeks, pts with CCR or near CCR were assigned to WW strategy while others underwent TME	307 (152-Induction arm, 155-Consolidation arm)	Primary- 3 year- DFS in each arm<br><br>Secondary- DFS, DMFS, Organ Preservation (OP) rates in each arm	Induction arm-<br>DFS-78%<br>DMFS-81%<br>OP-43%<br><br>Consolidation arm-<br>DFS-77%<br>DMFS-83%<br>OP-58%	In TNT setting, CRT followed by consolidation chemotherapy resulted in a significantly higher OP rate compared to induction chemo followed by CRT
**Fernandez-Martos et al, 2020-GEMCAD 1402 trial** [37]	Pts with LARC were randomly assigned (2:1), to mFOLFOX6 with (arm 1) or without Aflibercept (arm 2) prior to CRT and TME. Cases were classified as CMS-immune, epithelial or mesenchymal subtypes.	180 (arm 1-115, arm 2-65)	3 years DFS	Arm 1- DFS: 75.2%<br>Arm 2-DFS: 81.5% (p=0.56)<br><br>PCR rate was 27.5% and 0% in epithelial and mesenchymal subtypes respectively.	Adding aflibercept to induction mFOLFOX6 is not associated with an improvement in DFS. CMS subtype may predict PCR.
**Yuki et al, 2020-VOLTAGE trial** [34]	Studied use of nivolumab after CRT and before surgery in pts with LARC	42 (MSS-37, MSI-H-5)	PCR	MSS-30% PCR rate<br>MSI-H -60% PCR rate	Promising pCR rates of 30% and 60% were shown in MSS and MSI-H LARC pts treated with nivolumab plus

©2020 Kasi A et al. *JAMA Network Open*.

					radical surgery after CRT
**George et al, 2020-NRG-GI002: First experimental arm initial results [38]**	Pts with LARC were randomized to neoadjuvant FOLFOX (x 4mo) → CRT +/- veliparib 400mg PO BID → surgery 8-12 weeks later.	178 (88 control, 90 veliparib)	Primary- 4- point reduction in Neoadjuvant Rectal Cancer (NAR) score<br><br>Secondary- OS, DFS, toxicity, pCR, cCR, therapy completion, negative surgical margins, and SSS	Control vs Veliparib: Mean NAR score- 12.6 vs 13.7 (p=0.69)<br><br>PCR=21.6% vs 33.8% (p=0.14);<br><br>CCR=28.2% vs 33.3% (p=0.60);<br><br>SSS=52.5% vs 59.3% (p=0.43)	Veliparib added to CRT as a part of TNT was safe but did not improve NAR score
**Shamseddine et al, 2020 [36]**	Short-course radiation therapy (25 Grays in 5 fractions), followed by 6 cycles of mFOLFOX-6 plus Avelumab (anti PDL1), then total mesorectal excision (TME) in patients with LARC	13 out of the 44-pts accrued	PCR, major pathologic response, safety	PCR- 3/13 (25%)<br>Major pathologic response- 6/13 (50%)<br><br>No grade 3/4 AEs	Avelumab is tolerable when incorporated with short course RT as a part of TNT for LARC
**Romesser et al, 2020 [35]**	In Phase Ib (open), 18–30 pts with LARC will receive peposertib + capecitabine + RT<br>In Phase II, 150 pts will be randomized (1:1) to receive oral capecitabine + RT with either oral peposertib or placebo	Phase 1b- 18-30<br><br>Phase II- 150	Primary: To define maximal tolerated dose of peposertib, PCR, CCR<br><br>Secondary: Antitumor activity, safety, quality of life, pharmacokinetics of peposertib	Ongoing	Ongoing
**Capdevila et al, 2020-GEMCAD 1703 DUREC trial [33]**	Patients will receive 6 cycles of modified FOLFOX6 prior to	58	Primary: PCR	Ongoing	Ongoing

©2020 Kasi A et al. *JAMA Network Open*.

	CRT (capecitabine with 50.4 Gy in 28 fractions) and TME, combined with durvalumab 1500 mg every 4 weeks during induction CT, CRT and waiting period until surgery		Secondary: Toxicity, tumor regression grade, R0 resections, clear circumferential margins, surgical complications, NAR score, DFS and a biomarker program on tumor tissue, blood samples and stool microbiota		
**Fokas et al, 2019-CAO/ARO/AIO-12 trial** [39]	Patients with stage II or III rectal cancer were assigned to group A for induction chemotherapy using three cycles of fluorouracil, leucovorin, and oxaliplatin before fluorouracil/oxaliplatin CRT (50.4 Gy) or to group B for consolidation chemotherapy after CRT	306 (156 in group A and 150 in group B)	Primary: PCR<br><br>Secondary: Toxicity, compliance, and surgical morbidity.	Group A vs B<br>PCR= 17% vs 25%<br>Grade 3/4 toxicity= 37% vs 27%<br><br>Compliance to CRT: 91%, 78%, and 76% v 97%, 87% and 93% received full-dose radiotherapy, concomitant fluorouracil, and concomitant oxaliplatin in groups A and B, respectively<br><br>Compliance to chemotherapy:<br>92% vs 85%	Up-front CRT followed by chemotherapy resulted in better PCR, higher compliance with CRT but worse compliance with chemotherapy compared with induction chemotherapy followed by CRT

*PCR= Pathologic Complete Response, CRT- Chemoradiation, TNT-Total Neoadjuvant Therapy, DFS- Disease Free Survival, DMFS- Distant Metastasis Free Survival, OS- Overall Survival, MSI-H- Microsatellite Instability-High, MSS-Microsatellite Stable, LARC- Locally Advanced

©2020 Kasi A et al. *JAMA Network Open*.

Rectal Cancer, CCR- Clinical complete response, SSS- Sphincter sparing surgery, TME- Total mesorectal excision, CMS- Consensus Molecular Subtype

eFigure 1. Comparison of PCR Rates in TNT vs CRT Plus A (Meta-analysis of Nonrandomized Studies)

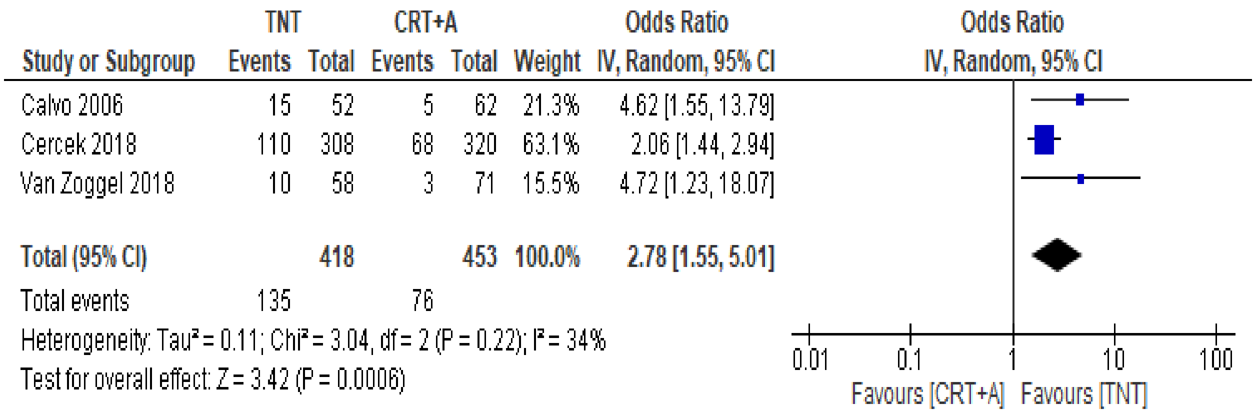

Fox plot comparing proportion of PCR between TNT and CRT+A [Non-randomized trials]. A random-effects model with inverse-variance method was used for the meta-analysis. Odds ratios are shown with 95% confidence interval.

©2020 Kasi A et al. *JAMA Network Open*.

eFigure 2. Comparison of PCR Rates in TNT vs CRT Plus A (Meta-analysis of Randomized Studies)

	TNT		CRT+A			Odds Ratio
Study or Subgroup	Events	Total	Events	Total	Weight	IV, Random, 95% CI
Conroy 2020	64	231	27	230	27.4%	2.88 [1.76, 4.72]
Garcia-Aguilar 2015	25	65	11	60	9.9%	2.78 [1.22, 6.34]
Markovina 2017	19	69	11	69	9.7%	2.00 [0.87, 4.61]
Van der Valk 2020	117	423	55	398	53.0%	2.38 [1.67, 3.40]
**Total (95% CI)**		**788**		**757**	**100.0%**	**2.51 [1.94, 3.25]**
Total events	225		104			

Heterogeneity: Tau² = 0.00; Chi² = 0.72, df = 3 (P = 0.87); I² = 0%

Test for overall effect: Z = 6.96 (P < 0.00001)

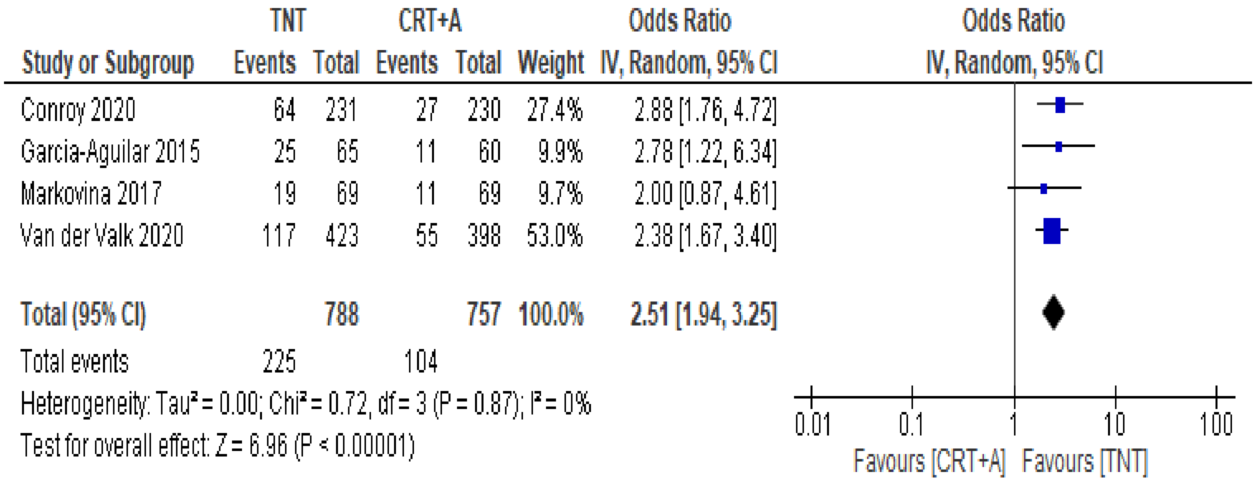

Fox plot comparing proportion of PCR between TNT and CRT+A [Randomized trials]. A random-effects model with inverse-variance method was used for the meta-analysis. Odds ratios are shown with 95% confidence interval.

©2020 Kasi A et al. *JAMA Network Open*.
